# Sub-area classification of environmental phosphorus loss risk in Yunnan’s crop-livestock systems: a spatiotemporal analysis

**DOI:** 10.1038/s41598-025-06302-4

**Published:** 2025-10-06

**Authors:** Xiaolin Li, Yanjie Wang, Jiecheng Wu, Lei Hou, Yi Zheng

**Affiliations:** 1 College of Plant Protection, Yunnan Agricultural University, Kunming, 650500 China; 2 College of Soil and Water Conservation, Southwest Forestry University, Kunming, 650224 China; 3 Department of Sustainable Development, Environmental Science and Engineering (SEED), KTH Royal Institute of Technology, 10044 Stockholm, Sweden; 4 Office of President, Yunnan Open University, Kunming, 650500 China; 5 College of Ecology and Environment, Southwest Forestry University, 650224 Kunming, China

**Keywords:** Crop-livestock system, Phosphorus, Sub-area classification, Nutrient flow, Environmental risks, Environmental impact, Agroecology

## Abstract

Crop and livestock production in Yunnan province has experienced significant growth over the last decade. However, this progress has come at the expense of excessive phosphorus (P) losses to the environment, leading to ecosystem degradation and adverse effects on human health. As a result, there has been a growing focus on mitigating P losses within the crop-livestock systems (CLSs). Nonetheless, research on quantitatively analyzing the spatial and temporal distribution of P flows in these systems remains inadequate. Addressing this issue requires adopting an appropriate spatial scale for P management, which balances accurate regional representation with sufficient data granularity. Using statistical data, literature and geospatial data collected from 1995 to 2014, a time series and spatial distribution of P flows associated with CLSs was analyzed using the SFA model (Substance Flows Analysis). The results showed that total P inputs to crop systems increased by 52% (from 2.89 × 10^5^ t in 1995 to 4.4 × 10^5^ t in 2014) in Yunnan province. Concurrently, feed import as a dominant input to animal subsystems experienced a minor decrease over the same period. Application of P fertilizer was the main source of P inputs. Soil accumulation emerged as a prominent pathway, contributing to 50% of the total P output in 2014. Additionally, losses to water, stemming from terrain conditions and improper fertilizer application patterns, represented other notable pathways. A spatial analysis of P use efficiency categorized the region into three sub-areas comprising 16 municipalities. In region I, soil testing and fertilizer regulation could support targeted P fertilizer application based on the nutrient supplying capacity of soil and crop nutrient demands. Region II calls for the integration of crop and livestock production, while the region III would necessitate the expansion of animal production and advancements in manure management technology. This approach of sub-area classification presents a promising strategy for improving nutrient management in areas with complex terrain and diverse climate condition.

**Supplementary Information:**

The online version contains supplementary material available at 10.1038/s41598-025-06302-4.

## Introduction

Phosphorus (P) plays a crucial role in supporting the growth of plants, animals and humans^1–5^. However, once released into the environment, excess P has led to negative impacts on the local ecosystem and biodiversity, including eutrophication, harmful algal blooms, oxygen depletion and fish kills^6,7^. In China, approximately 70% of P losses can be attributed to agricultural activities^8^. While fertilizer use has significantly increased, P uptake through crop harvest has not kept pace, resulting in an alarming accumulation in the soil of up to 39 kg P ha^− 1^year^− 1^ in crop production systems. In addition, although animal production also has greatly increased by 5 times in the two last decade, the amounts of P in animal manures have been largely neglected as a recycled nutrient to cropland in China^6,9^. This inappropriate use of animal manure and low efficiency of P cycling between livestock and crop-production systems has led to P leakage into aquatic systems^10^. To achieve agricultural production and water quality improvement, it is important to precisely quantify the losses of P from crop-livestock systems (CLSs).

An approach is needed to assess P flows between different compartments, such as crop and livestock production compartment. Models, spatially explicit ones, offer viable solutions to quantify and analyze P flows within a diverse range of CLSs. These models enable the execution of “virtual experiment”, which would otherwise be prohibitively expensive or impractical in real-world system^11,12^. For instance, the SWAT (Soil and Water Assessment Tool) model has been applied to watersheds to quantify the long-term impacts of potential changes in agricultural practices on river water quality^13,14^. The NAPI (Net Anthropogenic P Input) model was developed to estimate human-induced P inputs to drainage basins. FARMSIM model (African Smallholder Farm System) has been used to quantify nutrient flows among different agricultural production sectors^15^. However, these comprehensive models require large amounts of data for parameter fitting. Due to data scarcity in many regions of Yunnan province, collecting all the necessary data for these models are challenging and unrealistic.

Among the available models, the SFA (Substance Flows Analysis) model, of which the core principle is mass balance derived from the law of mass conservation, stands out as it involves quantifying nutrient transfers between species or sections within an agricultural ecosystem or a specific region^16,17^. Numerous studies have successfully utilized the SFA model to analyzed P flows at various spatial scales, including state, city, county and field^8,18–20^. However, relying solely on national average data may not adequately address regional questions, especially in Yunnan province with significant spatiotemporal variations in geographic factors, climate, soil, energy, and economic structure^21^. Moreover, a highly refined small-scale analysis is theoretically sound, but it also presents practical challenges. For example, collecting data for highly refined small-scale analysis was much more difficult and time-consuming than that at large scale. Therefore, to strike a balance between capturing regional heterogeneity effectively and ensuring sufficient data intensity, a proper spatial scale is essential. To address it, in this study, we used the sub-area scale to overcome the limitations of national average data and address regional question more accurately.

The general P cycle at national scale in China is reasonably well-understood. Wang provided a detailed analysis of P flow in the food chain in China and identified where P use efficiency can be improved, where P leaks from the food production system to the environment^22^. However, significant regional differences remain poorly quantified and comprehended in Yunnan province, particularly in areas with complex climatic and topographical conditions. Yunnan province is situated in southwestern China where the elevation decreases from the northeast to the southwest, resulting in substantial north–south climate variation. In western Yunnan, fertilizer application levels are much lower than in the eastern half. In certain areas, crop residues serve as animal feed or fuel, and animal manure is also used or fuel, potentially leading to soil nutrient depletion. However, estimates of P flows in CLSs and losses in Yunnan province at the sub-area scale are currently lacking.

The objective of this research presented in this paper was to assess the P flows, use efficiency and losses within CLSs at regional level and identify high environmental risk areas in Yunnan Province. To achieve this, we first applied the SFA model to analyze temporal distribution of P flows in the crop and livestock-production subsystems from 1995 to 2014. The year 1995 was selected as it marked the onset of rapid economic growth and agricultural development in Yunnan province, while 2014 represented the most recent year for which we had data at the beginning of this study. Next, we analyzed the spatial distribution of P flows, use efficiency and losses in crop and livestock-production subsystems at the municipality scale. Finally, we used sub-area classification to divide Yunnan’s 16 municipalities into three zones based on environmental risk, suggesting the implementation of distinct management actions in each zone. The research results have the potential to support the formulation of effective policies aimed at reducing P pollution and improving P management in agricultural production in the plateau area.

## Materials and methods

### Study area

**Fig. 1:**
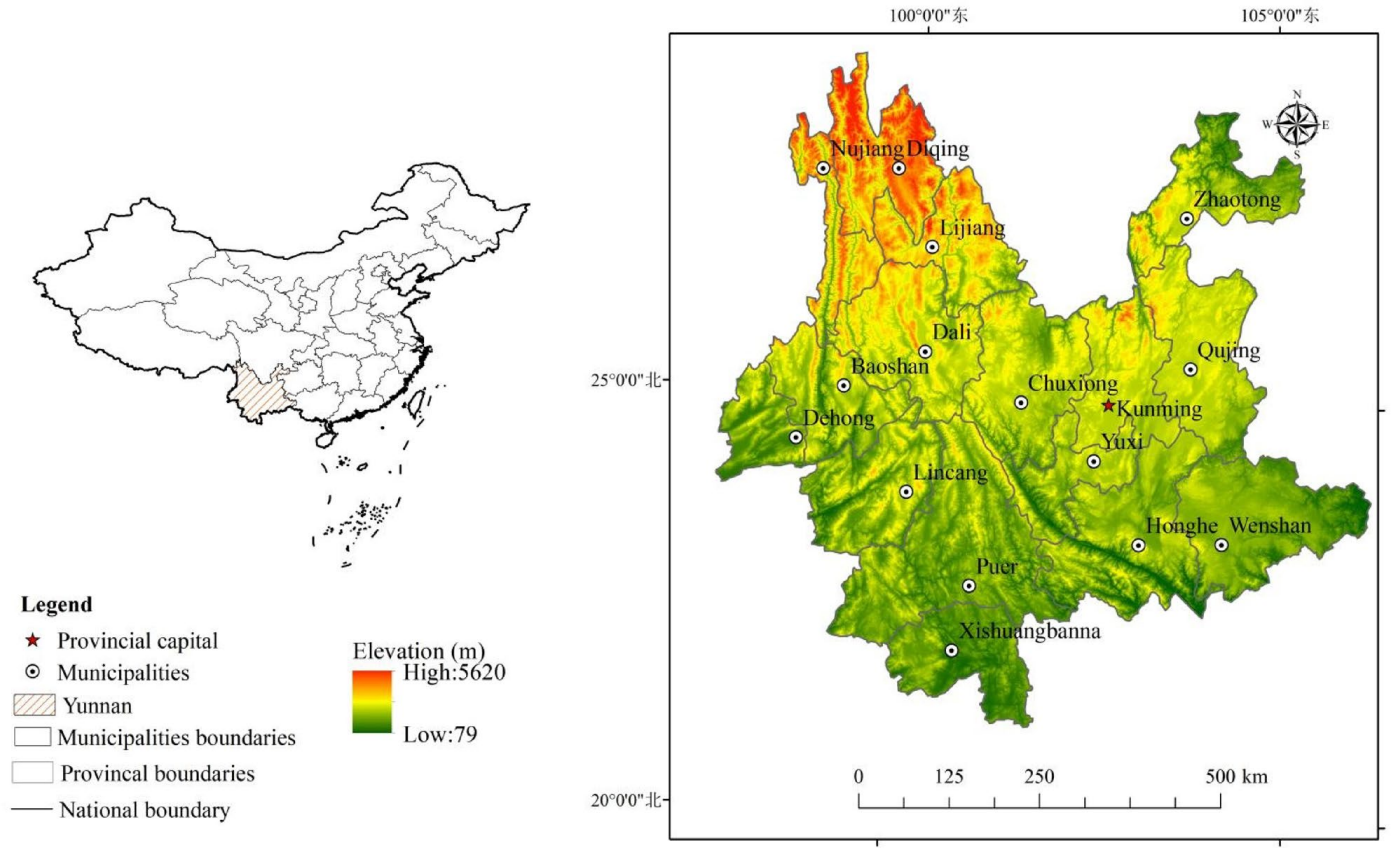
Location of Yunnan province and its elevation gradients. (By ArcGIS v.10.2.https://www.esri.com/en-us/arcgis/products/arcgis-desktop/overview/).

Yunnan province is located between 21°08′–29°15′ N, 97°32′–106°12′ E, sharing borders with Vietnam, Laos, and Burma (Fig. 1). Covering nearly 4.1% of China’s total areas (approximately 0.39 million km^2^), it ranks as the eighth-largest province in the country. The landscape is characterized by mountain areas, accounting for 84% of the territory, plateaus and hills occupying 10%, and basins and valleys making up only 6%^22^. Most regions in the northeast are at elevations greater than 2000 m, with an overall range of 1500 m to 4000 m, while the elevation of south-western area ranges from 800 m to 1000 m. The temperate continental monsoon climate of Yunnan Province shows substantial north–south differences. The region enjoys ample sunshine and abundant heat resources, with annual rainfall totaling 1100 mm, primarily concentrated during the summer^23^. The province contains 16 municipalities and 129 county-level administrative regions, with a total population of 4.7 million in 2014. The temporal and spatial distributions of crop and livestock production structures are shown in Fig. S1 and Fig. S2 in Supporting Information.

### SFA model

**Fig. 2:**
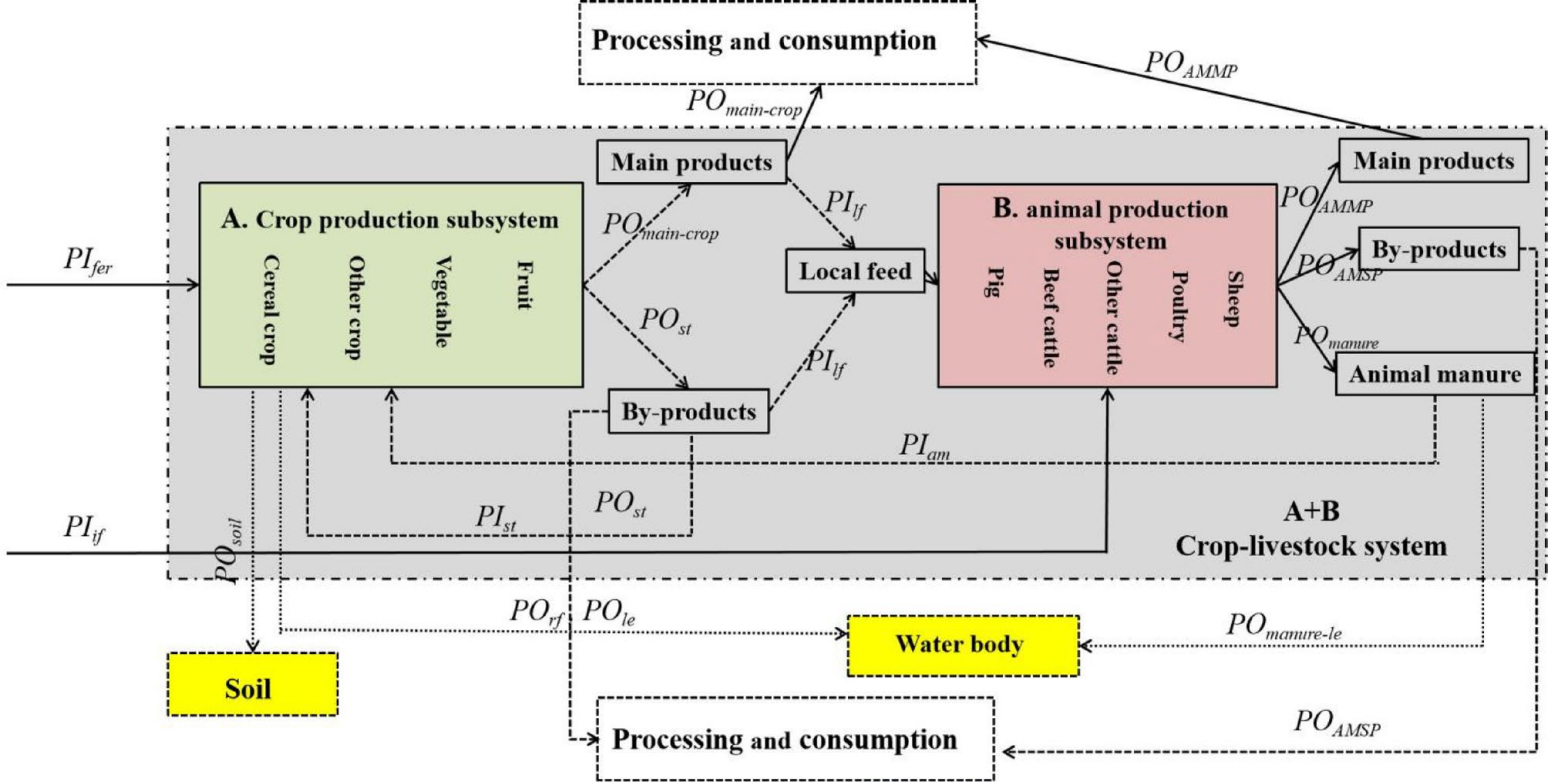
Demonstration of P flow model in crop-livestock production system (A + B, gray box) at municipality scale in Yunnan province, including crop production (A, green box) and animal production (B, red box). Inputs are shown on the left side. Solid arrow refers to the direction of P flow and parameter names are further explained in Table 1.

The SFA model was used to quantify the key stock and flows of P in the food chain system. The core principle of SFA is mass balance derived from the law of mass conservation, which has been used in past research to quantify P balance, losses, use efficiency, and the P cycle in various interconnected systems constituting the Chinese food chain, including crop production, animal production, food processing, and food consumption^16,17,24^. In this study, the SFA model was used to explore the characteristics of P flows in the CLSs of Yunnan province and to quantify P losses to soil and surface water. The whole process of P flows in the CLS considered was illustrated in Fig. 2. The CLSs of Yunnan province were examined, and the geographical boundaries of the 16 municipalities were defined as the study units. The SFA simulation covered the study period from 1995 to 2014, with the CLS of this model was composed of two main subsystems (sectors): crop production and animal production. In the crop-production subsystem, all types of crops, vegetables and fruits planted on arable lands were systematically considered. As for the animal production subsystem, food animals, including pigs, cattle, poultry, and sheep, were taken into account. Additionally, P flowed between the crop and livestock subsystems through the use of cropland products as animal feed or animal excreta as fertilizer on cropland. More details are provided in Sections A.1 of Supporting Information.The equations in Table 1 provide detailed information about the calculation method used in the model.

**Table 1:** Equation to quantify phosphorus efficiency (PUEa + c) and phosphorus loss (PL) from crop-livestock system by SFA model.

$\:{{PUE}}_{{c+a}}$	${{ = }}{{O_{{mainproduct}} } \mathord{\left/ {\vphantom {{O_{{mainproduct}} } {I_{{total}} }}} \right. \kern-\nulldelimiterspace} {I_{{total}} }}{{ \times 100}}$
$\:{O}_{mainproduct}$	$\:{=\,PI}{fer}{+\:PI}{if}{-PL}$
$\:{I}_{total}$	$\:{=\,}{\:PI}{fer}{+\:PI}{if}$
$\:{PL}$	$\:{=\,}{PO}{rf}{\:+PO}{le}{+PO}{manure-le}{+PO}{soil}$
Where,	
$\:{O}_{mainproduct}$	Output of *P* in main product from crop and animal production
*I_total*	Total *P* input from crop and livestock system
*PI*_*fer*_	Input of *P* via synthetic fertilizer including single and compound fertilizer
*PI*_*if*_	Input of *P* to animal production via feed from outside the municipality
*PO*_*main−crop*_	Output of *P* via main crop products from crop production
*PO*_*st*_	Output of *P* via straw from crop production
*PI*_*lf*_	Input of *P* to animal production via local feed
*PO*_*AMMP*_	Output of *P* via main animal products from animal production
*PO*_*AMSP*_	Output of *P* via animal by-products from animal production
*PO*_*manure*_	Output of *P* via animal manure
*PI*_*am*_	Input of *P* via animal manure application on land
*PI*_*st*_	Input of *P* via straw retuning to the land
*PO*_*soil*_	Output of *P* via accumulated in soil
*PO*_*rf*_	Output of *P* via runoff and erosion
*PO*_*le*_	Output of *P* via leaching
*PO*_*manure−le*_	Output of *P* via animal manure directly discharge into surface water

### Data and parameter sources

Various data and parameter sources were used to estimate P flows and losses in the CLSs. Data on synthetic fertilizer application, feed import, crop production, crop yield and animal number were extracted from the statistical yearbooks published by the Statistical Bureau of Yunnan province (Table S2, S3, S4 and S5 in Supporting Information). Additionally, published literature was used to obtain P contents of crop and animal products^8,20,25^ (Table S6 in Supporting Information). The annual P excretion per animal was calculated using the method reported by Ma^8^ (Table S7 in Supporting Information). Parameters pertaining to straw utilization, such as the ratio of straw to grain and P contents of crop products and straws, were derived from relevant literature and subsequently adjusted to suit Yunnan’s specific condition (Table S6 in Supporting Information). For the proportion of crop residues used as feed and animal manure returned to cropland, empirical factors were applied, derived from field surveys and direct interactions with local experts (Table S8, S9, S10 and S11 in Supporting Information). ArcGIS (version 10.2) was utilized to analyze the spatial distribution of P flows and associated environmental factors. Detailed information about the data and parameter sources is provided in Supporting Information Table S1. More details are provided in Sections A.2 of Supporting Information.

## Results

### Temporal distribution of P flows in the crop and livestock-production subsystems

**Fig. 3:**
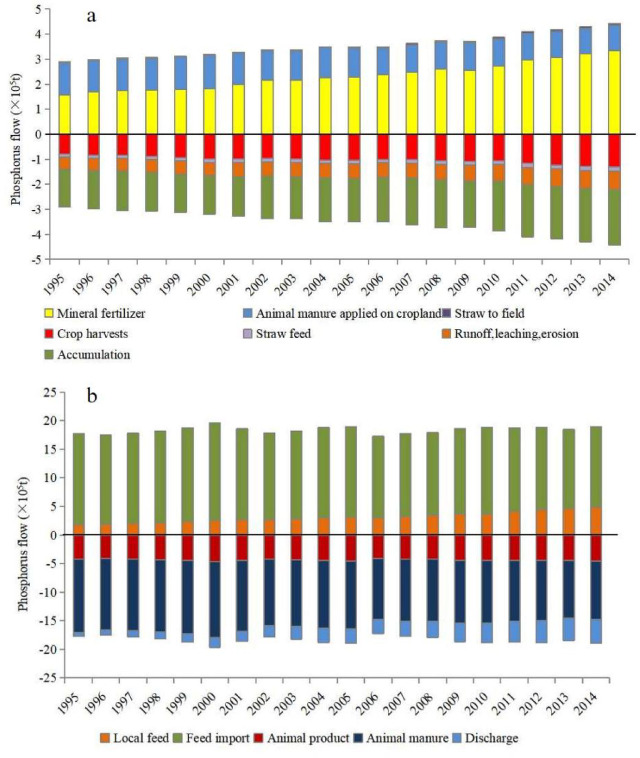
Phosphorus flow in crop (**a**) and livestock (**b**) production systems from 1995 to 2014 in Yunnan province. Positive and negative dimension in X-axes indicate phosphorus input and output items in crop-livestock systems, respectively.

The variation in P flows within the crop-production subsystem from 1995 to 2014 is illustrated in Fig. 3a. The total annual P inputs to cropland increased by 52% (from 2.89 × 10^5^ t in 1995 to 4.4 × 10^5^ t in 2014). Notably, the predominant inputs to the crop-production subsystem were attributed to synthetic fertilizer, which increased from 1.58 × 10^5^ t (constituting 55% of the total P input) in 1995 to 3.34 × 10^5^ t (76%) in 2014. Conversely, the contribution of P from animal manure declined from 1.28 × 10^5^ t (44%) in 1995 to 1.02 × 10^5^ t (23%) in 2014. Among the various pathways of P outputs, crop harvest emerged as dominant, constituting 25% of total P outputs. Soil accumulation was the dominant source of P losses, accounting for 2.21 × 10^5^ t in 2014 and constituting 50% of total P output. Furthermore, P losses via runoff, leaching, and erosion underwent a 1.5-fold increase from 1995 to 2014.

The fluctuation in P flows within the livestock-production subsystem is depicted in Fig. 3b, indicating slight oscillations in P input and output levels over the two decades. Local feed, a minor P input component (contributing only 10–26% of total inputs), exhibited a modest increase, while feed imports experienced a minor decrease during the period. The export of P through manure, constituting the largest P output component, decreased from 1.28 × 10^5^ t in 1995 to 1.02 × 10^5^ t in 2014. Conversely, the amount discharged (i.e., not collected or managed in any facility and presumably released into the environment) underwent an increase from 0.07 × 10^5^ t in 1995 to 0.42 × 10^5^ t in 2014. Moreover, animal product accounted for approximately 24% of P outputs and remained relatively stable through the research period.

### Spatial distribution of P flows in crop and livestock-production subsystems

**Fig. 4:**
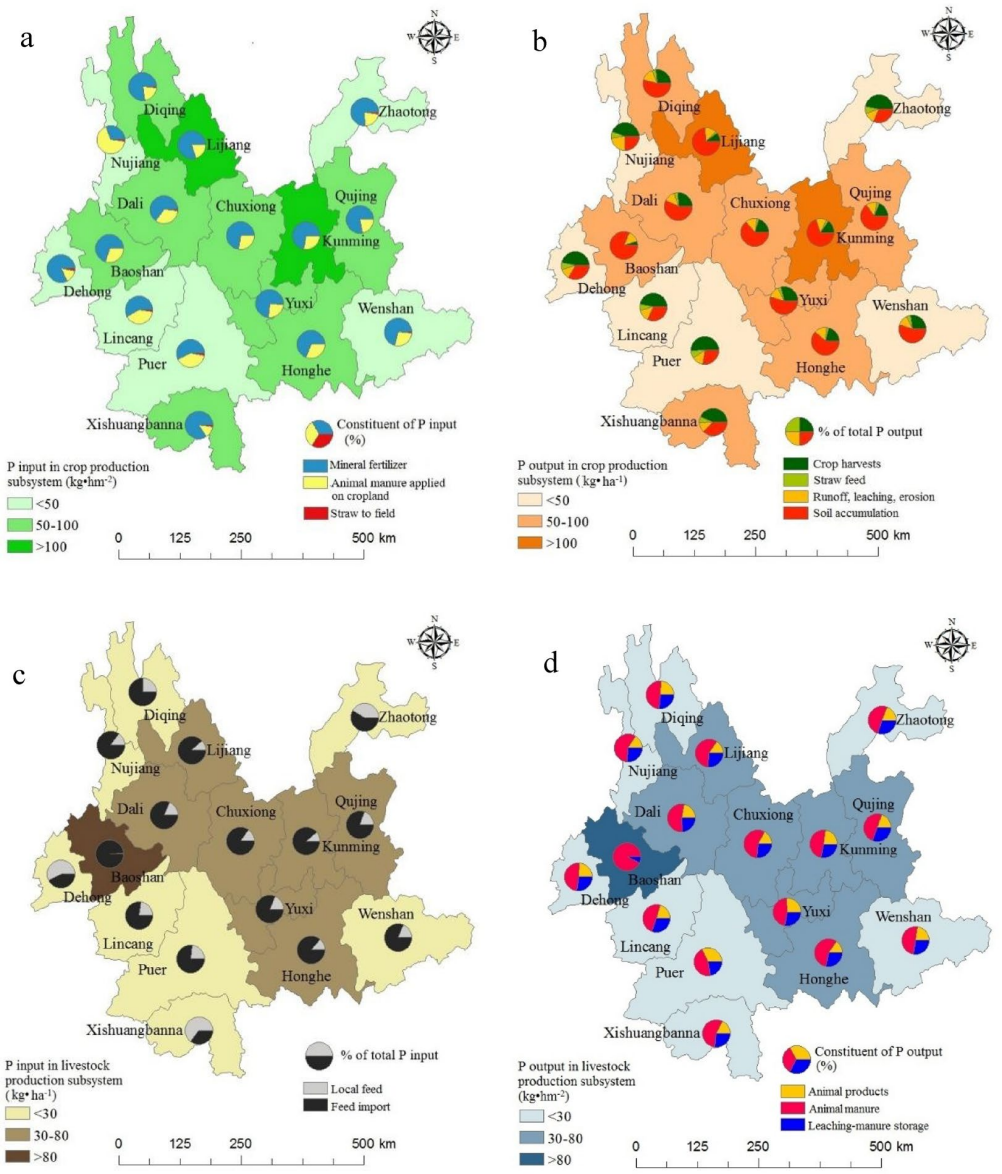
spatial distribution of phosphorus inputs (**a**) and outputs (**b**) in crop production subsystem, inputs (**c**) and outputs (**d**) in animal production subsystem in 2014 (By ArcGIS v.10.2.https://www.esri.com/en-us/arcgis/products/arcgis-desktop/overview/).

Spatial variation in P inputs within the crop-production subsystem for the year 2014 is presented in Fig. 4a. Yunnan province was categorized into three regions based on P input levels. As shown in Fig. 4a, Kunming and Lijiang (the first region) exhibited inputs exceeding 100 kg ha^– 1^. The second region, encompassing P inputs in the crop subsystem ranging from 50 kg ha^– 1^ to 100 kg ha^– 1^, covered under 49% of the total land areas yet accounted for 62% of total P inputs for crop production in Yunnan during 2014. Regions with lower P input levels (< 50 kg ha^– 1^) were dispersed across eastern and southwestern Yunnan province. According to P outputs from the crop-production subsystem, the 16 municipalities of Yunnan province were classified into three regions with different sources (illustrated in Fig. 4b). A comparable spatial trend was evident for P inputs and outputs associated with the crop subsystem in 2014. In regions with P outputs exceeding 100 kg ha^– 1^, soil accumulation outstripped runoff, leaching, and erosion as a significant P output pathway. Conversely, in areas with outputs below 50 kg ha^– 1^, crop harvest emerged as the predominant P output.

Based on P inputs to the animal production subsystem, the 16 municipalities of Yunnan province were partitioned into three regions (shown in Fig. 4c). The first region, with P inputs surpassing 80 kg ha^– 1^, solely encompassed the municipality of Baoshan. The second region, with P inputs ranging from 30 kg ha^– 1^ to 80 kg ha^– 1^, included 7 municipalities. The remaining 6 municipalities constituted the third region, with P inputs below 30 kg ha^– 1^. Notably, feed import stood as the principal source of P, contributing to approximately 70% of the total P inputs to animal production for most municipalities. Dehong and Xishuangbanna deviated slightly, with feed import accounting for 42% and 35%, respectively. The spatial variation in P outputs mirrored the input pattern for the livestock-production subsystem in 2014 (depicted in Fig. 4d). P outputs linked to livestock production spanned from 133.4 kg ha^– 1^ in Baoshan to 10.9 kg ha^– 1^ in Dehong. Manure export constituted the primary form of P outputs, representing over 45% of the total outputs across all 16 municipalities of Yunnan province.

### Spatial distributions of P inputs and losses in CLS

**Fig. 5:**
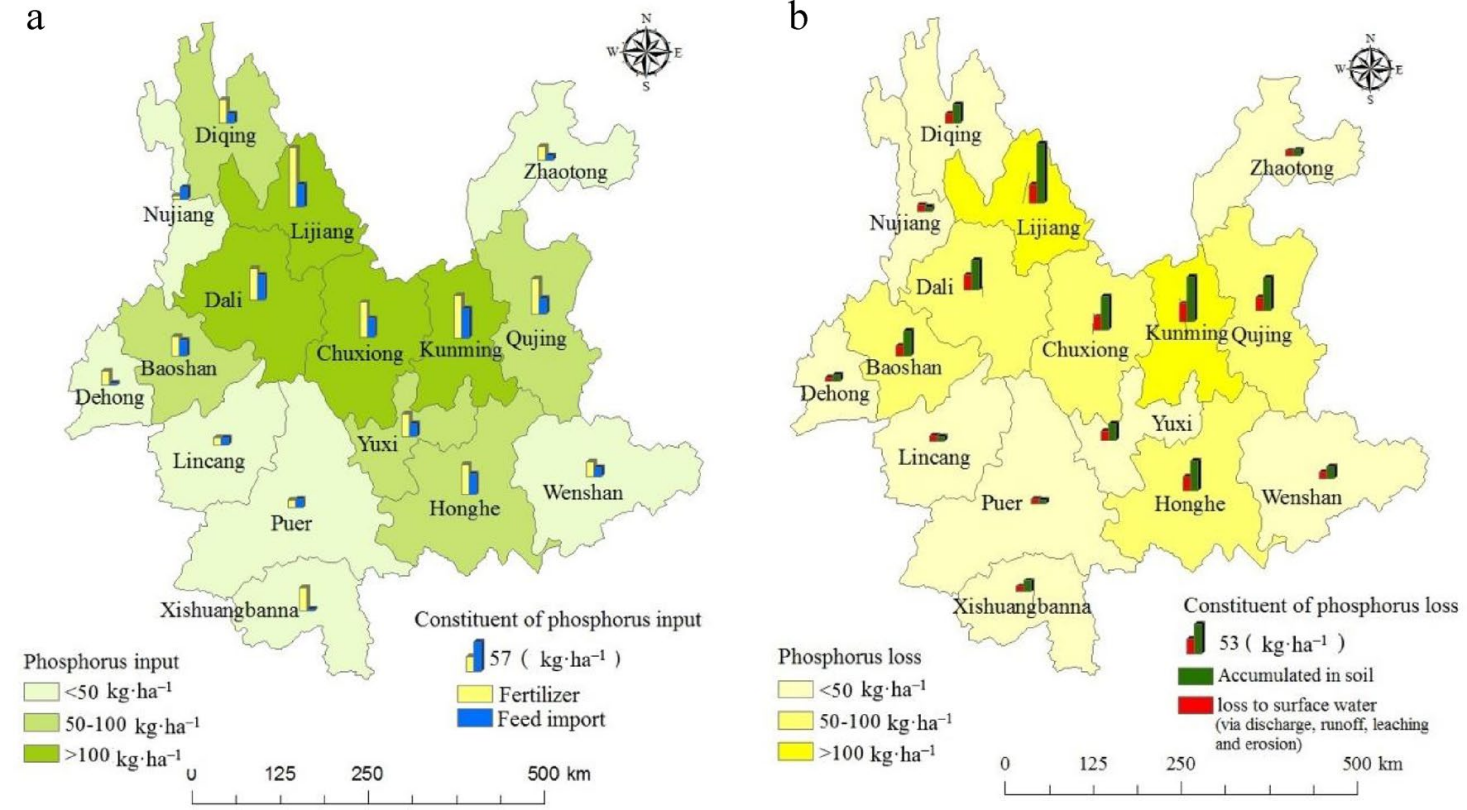
Distribution map of phosphorus input (**a**) and losses (**b**) in CLS in 2014 (By ArcGIS v.10.2.https://www.esri.com/en-us/arcgis/products/arcgis-desktop/overview/).

Based on the P flows trends in crop and livestock subsystems described above, the distributions of P inputs and losses within the CLSs of the 16 municipalities in 2014 are shown in Fig. 5. The total P inputs displayed notable variation among municipalities, with P inputs per unit area of arable land spanning from 30 kg ha^– 1^ (in Lincang) to 158 kg ha^– 1^ (in Lijiang). In central Yunnan province, P inputs per unit area of arable land was consistently elevated, exceeding 100 kg ha^– 1^, largely attributed to substantial fertilization and feed imports.

In the year 2014, the total P losses was estimated at 2.83 × 10^5^ t, accounting for 64% of the total P inputs. The spatial distribution of P losses across the 16 municipalities is depicted in Fig. 5b. P losses were classified into three levels, with the highest loss category (exceeding 100 kg ha^– 1^) observed in Kunming and Lijiang. Within a single municipality, the contribution of soil accumulation to total P losses outweighed that of runoff, leaching, and erosion. In Lijiang, P losses attributed to soil accumulation reached 106 kg ha^– 1^, constituting over 76% of the total, while losses to surface water was estimated at a mere 32.8 kg ha^– 1^.

### Sub-area classification of P environmental risk

PUEa + c, a widely used metric for both policymakers and farmers, serves as a tool to gauge the potential risk of P losses. Essentially, 1- PUEa + c estimates the potential P losses to the environment as a proportion of the P inputs, thus serving as an indicator of the associated P use risk. The average PUE for the CLSs in Yunnan Province was 29.6% in 2014. PUEa + c decreased from 31.5% in 1995 to 25.6% in 2014, with the recycling of P from animal manure in crop production decreased dramatically. The animal manure produced contains most of P originating from the feed, but the economic cost of manure transport to cropland is found to be too high, which resulting in a large fraction of manure from larger-scale industrial farming dumped into landfills or discharges into surface water almost untreated.

**Fig. 6:**
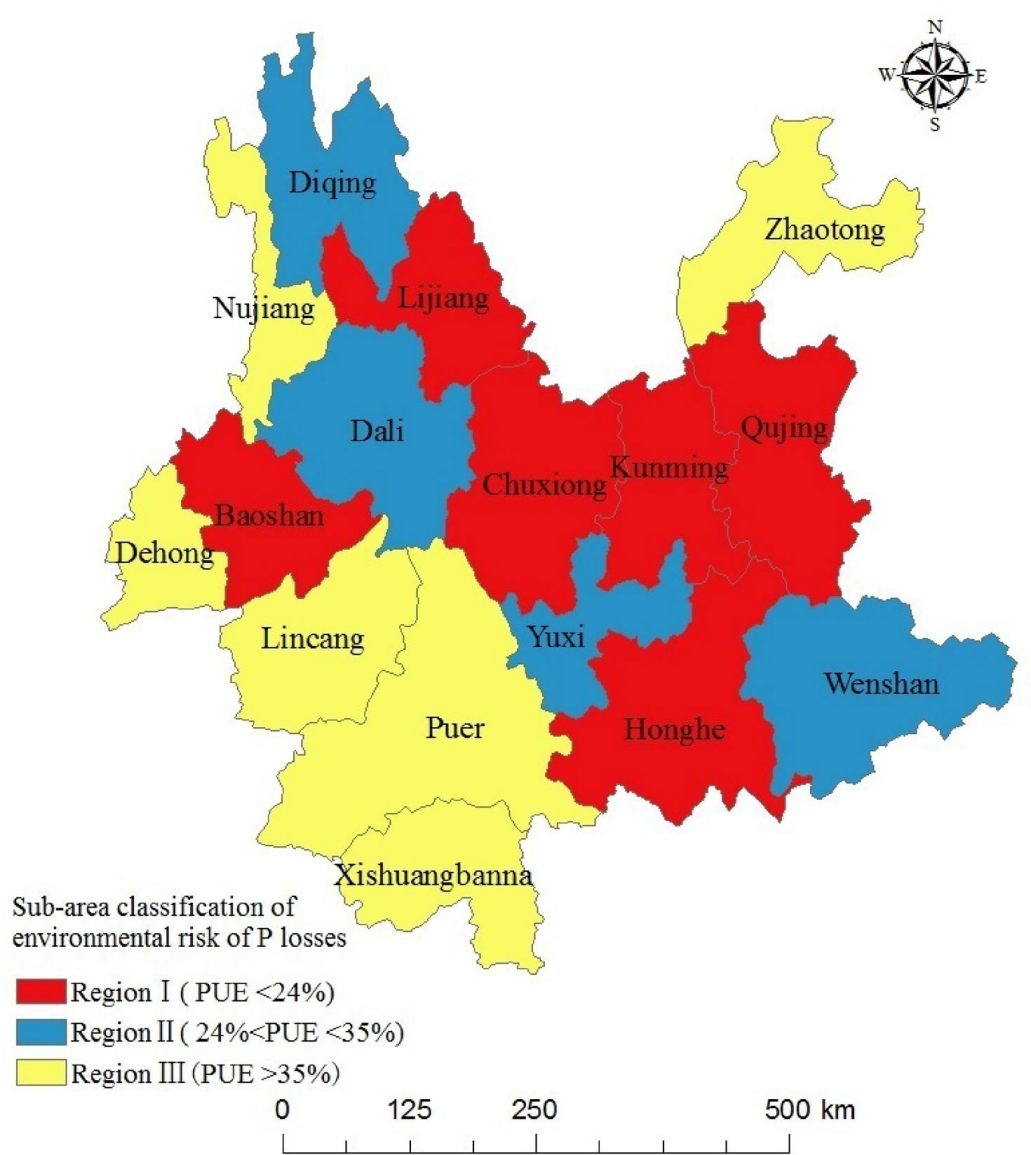
Sub-area classification of environmental risk of P losses and PUE (By ArcGIS v.10.2.https://www.esri.com/en-us/arcgis/products/arcgis-desktop/overview/).

Utilizing the national-level average PUEs for the CLSs in 2005 (35%) and 2013 (24%), we classified the 16 municipalities into three zones based on PUE values (as depicted in Fig. 6). Zone I encompassed municipalities with a lower PUE (< 24%), including Kunming, Qujing, Lijiang, Baoshan, Chuxiong, and Honghe, characterized by relatively higher prosperity. Zone II featured a median PUE (> 24% and < 35%), comprising Yuxi, Wenshan, Dali, and Diqing. Lastly, zone III, characterized by a high PUE (> 35%), included Zhaotong, Pu’er, Lincang, Xishuangbanna, Dehong, and Nujiang. Region III was predominantly characterized by crop production. More details about municipality-level PUE values and risk categories can be found in Table S12 in Sections A.3 of Supporting Information.

## Discussion

### Uncertainty of the results

Our model has uncertainties associated with model input and parameters. For example, we derived many model inputs for agricultural activities (e.g. synthetic fertilizer application, the production of different kinds of crops, feed import) from the Yunnan statistical yearbook (see Table S1). This statistical yearbook provides inputs for 129 counties in Yunnan province. We merged county-scale inputs to municipality-scale input by an area-weighted method in ArcGIS. And statistical yearbook data may under reporting the use of animal manure. Furthermore, some model parameters were taken from the NUFER model that was validated for 31 provinces in China, including Yunnan province. These are, for example, the emission factor of P from animal production via animal manure leaching during storage (see Table S11), which will lead to uncertainty in the calculated P losses to the waters. In addition, there is a limitation of SFA model which cannot capture dynamic interactions between subsystems. Nevertheless, this should not create any large deviations because the main contributors to P loss and P management are consistent with the local agricultural activities of Yunnan province. Uncertainties in the model were addressed by comparing the model outputs with those of past studies. Our estimated PUE for the CLSs in Yunnan Province was 29.6% in 2014, which is consistent with the findings of Chen^23^. In terms of P losses, our findings underscore that soil accumulation constitutes the primary route of P losses, aligning with the conclusion of Ma^8^. Although there are also some differences with other previous studies, they could be explained reasonably. For example, our result is lower than the average values observed in the United Kingdom (56%), as reported by Withers^26^, and in Finland (46%)^27^, as estimated by Antikainen. Our estimated PUE in Yunnan province was also lower than that in other regions in China, such as Beijing^8^ and Haihe basin^28^. This pattern underscores the inefficiency in P inputs for crop and animal production within Yunnan province due to inadequate nutrient management within CLSs. This pattern underscores the inefficiency in P inputs for crop and animal production within Yunnan province due to inadequate nutrient management within CLSs. The proportion of total P losses attributed to soil accumulation (52%) surpasses Ma’s estimation (46%)^8^. This difference could be attributed to the acidic soil nature of Yunnan province, particularly evident in certain western regions where soil pH dips below 6.5. In acidic soil conditions, organic P can react with iron, forming insoluble compounds that tend to accumulate within the soil matrix.

### Temporal and spatial distribution of P flow in CLSs

The inputs of P to CLSs exhibited a noteworthy escalation during the period from 1995 to 2014. Specifically, P inputs to crop systems increased from 2.89 × 10^5^ t, while inputs into the animal production subsystem experienced fluctuations from 1.77 to 1.89 × 10^5^ t over the preceding two decades. Three main factors have driven the increasing P cost of agricultural production. Firstly, the imperative to sustain the burgeoning Chinese population’s food demands has prompted governmental incentives (subsidies) encouraging the utilization of nitrogen (N) and P fertilizers to enhance crop yields. Predominantly, mineral fertilizer served as the main P source, witnessing an upward trend from 1.58 × 10^5^ t in 1995 to 3.34 × 10^5^ t in 2014. Secondly, the escalation in the area allocated to cultivating vegetables and fruits played a role. Since the implementation of the “Shopping Basket Program” in the 1990s, Yunnan province has emerged as a pivotal hub for vegetable and fruit production within China^28–30^. The sown areas for vegetables expanded by a remarkable 4.5-fold, while that for fruits witnessed a 2.8-fold increase- figures surpassing other crop types. Evidently, P inputs from fertilizer into vegetable and fruit production systems are exceptionally high. Thirdly, the rapid expansion of livestock farming has further fueled this increase. Amplified consumption of animal-derived products has driven a twofold surge in livestock units over less than two decades, propelled by the growth of highly intensive industrial livestock systems and monogastric livestock. The P content in imported feed constituted a substantial share of total nutrient inputs into agriculture, which is in line with the findings from other P flows analyses conducted across China.

The P flows within the crop-production subsystem in Yunnan province showed significant spatial heterogeneity, with P input per unit of cultivated land area reflects the regional economic development. Similar relationships have been identified in other countries^31^. Regions exhibiting relatively elevated prosperity, exemplified by Kunming (reaching 144.1 kg ha^– 1^) and Lijiang, correlate with heightened P inputs. Yunnan province, in recent decades, has delineated specific zones for livestock production through policy directives, fostering distinct spatial patterns in livestock farming; cattle predominant in the west, poultry in the central region, and pigs in the northwestern region. This flourishing livestock industry has led to relatively elevated P inputs from animal feed, as evident in Baoshan, where it reaches 133.4 kg ha^– 1^. By contrast, regions characterized by crop production, such as Nujiang and Dehong, exhibit comparably lower input levels. Geologically and climatically distinct, Nujiang’s P inputs were among the lowest in Yunnan province, standing at a mere 24.5 kg ha^– 1^ due to its lower social development level among municipalities. Western Dehong, attributed with relatively diminished livestock production, recorded a P input of only 10.9 kg ha^– 1^. However, while these policies promote the development of livestock production, they fail to account for the necessary supporting farmland to accommodate animal manure generated during the breeding process. As a result, a large fraction of the manure is dumped in landfills or discharge into surface water without proper treatment, rather than returned on the cropland as organic fertilizer.

### Sub-area classification of environmental risks of P losses and PUE in CLSs

Soil accumulation and losses to surface water, via discharge, runoff, leaching, and erosion, stand as the primary contributors to P losses within CLSs. Around 20% of this considerable P loads accumulate within cropland soils (covering 6.21 × 10^6^ ha), leading to an average P surplus of 35.6 kg P ha^– 1^ year^– 1^. This surplus significantly exceeds the China-wide average of 28 kg P ha^– 1^ year^– 1^^20^. And it nears the upper limits noted in various developed countries for annual P accumulation in agricultural soils, with, for instant, an average of 20 kg P ha^– 1^ year^– 1^ in Europe^32^, 16 kg P ha^– 1^ year^– 1^ in the United Kingdom, approximately 30 kg P ha^– 1^ year^– 1^ in France, and 25 kg P ha^– 1^ year^– 1^ in Germany^26^. The regional variance in soil P accumulation emerges from cropping practices. Across the study span, although Kunming’s total cultivated area remained relatively constant, there was a shift from cereals to vegetables and fruits (Fig S1), necessitating higher mineral fertilizer application rates for vegetables and fruits compared to cereals. PUEa + c decreased from 31.5% in 1995 to 25.6% in 2014, and P losses to water increased from 0.49 × 10^5^ t in 1995 to 0.74 × 10^5^ t in 2014, with direct discharge of animal manure emerging as the most prominent factor. The reason might be that the transition from mixed crop–livestock production systems to landless animal-production systems during 1995–2014^33–36^. As a consequence, the recycling of P from animal manure in crop production decreased dramatically during this period. The animal manure produced contain most of P originating from the feed, but the economic cost of manure transport to crop land is found to be too high, which resulting in that a large fraction of manure from larger scale industrial farming dumped in landfills or discharges into surface water almost untreated. This regional variation is linked to dominant animal species. Xu et al. identified higher environmental pollution risk from pig production compared to other livestock categories^37^. Qujing in Yunnan province experiences elevated levels of P losses to aquatic systems due to extensive pig farming.

PUE has commonly served policymakers and farmers as an indicator of P loss risk. A value of 1 – PUE denotes the potential P losses to the environment relative to the P inputs and hence is used as a gauge of P risk. Based on environmental risk, we categorized Yunna’s 16 municipalities into three zones. Region I, marked by elevated crop and animal-based food production and consumption, exhibits higher prosperity and lower PUE. Furthermore, landless industrial animal-production systems predominate in this zone. These systems, larger and distinct from cropland, often face challenges in economically transporting manure to cropland. Consequently, a significant portion of the manure produced is either deposited in landfills or released into surface waters untreated. In contrast, region III, focused on crop production, demonstrates relatively high PUE. This region predominantly adopts traditional mixed smallholder farming systems where livestock and cropland coexist. Integrating crop and livestock production allows for effective reduction of manure discharge into water bodies by transforming livestock waste into crop fertilizer.

To achieve effective pollution reduction, we recommend partitioning Yunnan province into three sub-areas according to environmental risks, each undergoing tailored management strategies. In region I, where P fertilizer has been over-used, surpassing P uptake by harvested crops, soil testing and fertilizer programs could help to limit P fertilizers application based on the nutrient-supplying capacity of the soil and crop nutrient demands. Lime incorporation presents a valuable strategy for augmenting PUE through soil pH adjustments. In region II, integration of crop and livestock production is essential. Cutting down on feed imports, substantial components of CLSs inputs, can be achieved by enhancing local feed utilization through crop-livestock integration. Animal manure can then substitute mineral fertilizer when recycled as crop fertilizer, shifting P from the livestock subsystem to the crop subsystem. Region III necessitates the expansion of animal production and advancements in manure management technology. Addressing food production needs and population growth requires the establishment of large-scale industrial animal production systems to replace traditional mixed smallholder farming. Critical infrastructure investments in industrial animal production infrastructure are imperative to prevent P losses, facilitating proper collection, storage, and transport of manure to nearby cropland.

### Method applicability and future research opportunities

Assessing the spatial and temporal distribution of P environmental risk holds valuable insights for promoting sustainable nutrient management in agricultural production. Historically, the evaluation of P risk in CLSs has been confined to specific time points and administrative regions, with only a limited number of studies delving into P risk changes over extended time frames. Consequently, P management strategies have focused on predominantly on the present, often omitting the consideration of sustainable developmental needs. In this study, a spatiotemporal analysis of environmental P emissions across Yunnan province’s 16 municipalities during 1995–2014 was undertaken, presenting a fresh outlook on regional nutrient management and offering a comprehensive assessment of nutrient flows. The final results provide a basis for sustainable P management in the future.

Sub-area classification can improve nutrient management in regions characterized by intricate terrain and climate dynamics. Traditional studies of P flows have largely focused on the national, provincial and county scales. However, in regions marked by intricate topographical and climatic conditions, averaging outcome at the national or provincial level fails to capture the spatial diversity inherent in P flows. For example, this study demonstrates that in central Pu’er, Yunnan province, PUE reached an impressive 51.78%, whereas Baoshan was lower PUE to 11.22%. In contrast, Ma et al. determined PUE for Yunnan province at provincial level to be 36%^8^. If the provincial findings were applied as a guide for P management, regional strategies would not effectively address regional disparities. Additionally, county-scale calculations demand extensive data and parameter estimations, a task that becomes notably complex in regions characterized by intricate terrains. Therefore, this study proposes a novel sub-area management approach, enhancing resolution without necessitating the collection of the large number of parameters required for county-scale calculation. This innovation substantially augments the model’s efficiency.

## Conclusion

This study quantified historical trends in P inputs, outputs and flows within CLSs across Yunnan province. It is the first study to analyze the temporal (1995–2014) and spatial (16 municipalities of Yunnan province) variabilities in P use efficiency using SFA model. This sub-area classification approach offers a promising strategy to enhance nutrient management in regions characterized by complex terrain and varying climatic condition.

Our results are summarized as follows:

1. Total P inputs to crop systems in Yunnan Province increased by 52%, whereas inputs to animal subsystems fluctuated during the same period. The application of P fertilizer emerged as the predominant source of input. which enhances resolution without the arduous task of amassing an extensive set of parameters necessary for county-scale calculations. This development substantially bolsters the model’s efficacy. The principal sources of P losses were soil accumulation and losses to surface water through discharge, runoff, leaching, and erosion. The shift from mixed crop–livestock production systems to landless animal-production systems played a pivotal role in escalating P losses to water bodies, as the latter offer limited scope for recycling animal manure to support crop production.
2. The 16 municipalities of Yunnan were classified into three distinct zones based on environmental risk. Region I encompassing Kunming, Qujing, Lijiang, Baoshan, Chuxiong, and Honghe, featured low PUE (< 24%). This region boasts high levels of crop and animal-based food production and consumption. Region II had median PUE (> 24% and < 35%) (Yuxi, Wenshan, Dali, and Diqing). Zone III, comprising Zhaotong, Pu’er, Lincang, Xishuangbanna, Dehong, and Nujiang, exhibited high PUE (> 35%). This region predominantly accommodates traditional mixed smallholders who meld animal production with cropland.
3. Different management strategies are advocated for each region. For region I, soil testing and fertilizer programs could help to limit P fertilizer application. In region II, integration of crop and livestock production is advised. In region III, a proactive approach involves scaling up animal production and enhancing manure treatment technology.

In conclusion, our study provides a novelty way to maintain agricultural production (Global Sustainable Development Goals (SDGs) 2 “Zero Hunger”) while ensuring low water pollution (SDG 6 “Clean Water and Sanitation”). The study emphasizes the necessity of addressing P losses from CLSs to prevent environmental risk. The utilization of the SFA model and spatial analysis provides valuable insights into P flows distribution, allowing for specific management recommendations tailored to distinct regions within the province. Such sub-area classification approach also can be applied to improve agricultural nutrient management in other regions with intricate topography and diverse climatic conditions.

## Electronic supplementary material

Below is the link to the electronic supplementary material.

Supplementary Material 1.

## Data Availability

Access to the dataset could be available from the corresponding authors upon reasonable request.
