# Supplementary material for: Sub-area classification of environmental phosphorus loss risk in Yunnan’s crop-livestock systems: a spatiotemporal analysis
Source: Sci Rep. 2025 Oct 6;15:34678. doi: 10.1038/s41598-025-06302-4 (PMC12501268; doi:10.1038/s41598-025-06302-4)
Supplement: Supplementary file 1 — Supplementary Material 1. [file 41598_2025_6302_MOESM1_ESM.docx]

**Supporting Information**

**Sub-area Classification of Environmental Phosphorus Loss Risk in Yunnan’s Crop-Livestock Systems: A Spatiotemporal Analysis**

Xiaolin Li ^1,2^, Yanjie Wang ^2^, Jiecheng Wu ^3^, Lei Hou ^2^, Yi Zheng ^1,4,*^

1. College of Plant Protection, Yunnan Agricultural University, Kunming650500, China
2. College of Ecology and Environment, Southwest Forestry University, Kunming650224, China
3. Department of Sustainable Development, Environmental Science and Engineering (SEED), KTH Royal Institute of Technology, SE-100 44, Stockholm, Sweden
4. Office of President, Yunnan Open University, Kunming 650500, China

- Corresponding author: Yi Zheng Email: zhengyi@ynou.edu.cn (Y. Zheng)

A.1 Model description

We developed the Substance Flows Analysis (SFA) model that uses a mass balance approach to quantify P balance, losses, use efficiency, and the P cycle between crop production and animal production at municipalities scale in Yunnan province, southwest of China. The model simulation covered the study period from 1995 to 2014, with the crop-livestock system (CLS) of this model was composed of two main subsystems (sectors): crop production and animal production. In the crop-production subsystem, all types of crops, vegetables and fruits planted on arable lands were systematically considered. As for the animal production subsystem, food animals, including pigs, cattle, poultry, and sheep, were taken into account. Additionally, P flowed between the crop and livestock subsystems through the use of cropland products as animal feed or animal excreta as fertilizer on cropland.The equations provide detailed information about the calculation method used in the model as follows:

| $\text{PUE}_{\text{c+a}}\text{=}O_{mainproduct}\text{/}I_{total}\text{×}\text{100}$ | $\text{（}\text{1}\text{）}$ |
| --- | --- |
| $O_{mainproduct}\text{=PI}\text{fer}\text{+ PI}\text{if}\text{-PL}$ | $\text{（}\text{2}\text{）}$ |
| $I_{total}\text{=}\text{ PI}\text{fer}\text{+ PI}\text{if}$ | $\text{（}\text{3}\text{）}$ |

Where $\text{PUE}_{\text{c+a}}$ is phosphorus efficiency from crop-livestock system (%);$O_{mainproduct}$ is output of P in main product from crop and animal production, $I_{total}$ is total P input from crop and livestock system, $\text{PI}\text{fer}$ is input of P via synthetic fertilizer including single and compound fertilizer (kg).$\text{ }\text{PI}\text{if}$ is input of P via feed (kg). $\text{PL}$ is phosphorus loss from crop-livestock system (kg).

| $\text{PL}\text{=}\text{PO}\text{rf}\text{ +PO}\text{le}\text{+PO}\text{manure-le}\text{+PO}\text{soil}$ | $\text{（}\text{4}\text{）}$ |
| --- | --- |

Where $\text{PO}\text{rf}$ is output of P via runoff and erosion (kg),$\text{ PO}\text{le}$ is output of P via leaching (kg), $\text{PO}\text{manure-le}$ is output of P via animal manure directly discharge into surface water (kg). *PO_soil_* is output of P via accumulated in soil (kg).

| $\text{PO}\text{rf}$*=(PI_fe_+PI_st_+PI_am_)*$\text{×}$*EF_runoff and erossion_* | $\text{（}\text{5}\text{）}$ |
| --- | --- |
| *PI_s_=Prod_crop_×Rat_straw_*$\text{×}$*Pcon_straw_*$\text{×}$*Rat_straw-field_* | $\text{（}\text{6}\text{）}$ |
| *PI_am_=*$\text{NU}\text{ani}\text{×}$*Pex_ani_*$\text{-}\text{PO}\text{manure-le}$ | $\text{（}\text{7}\text{）}$ |

Where *PI_st_* is input of P via straw retuning to the land (kg), *PI_am_* is input of P via animal manure application on land (kg), *EF_runoff and erossion_* is emission factor of P from crop production by runoff and erossion. *Prod_crop_* is the production of different kinds of crop (maize, wheat, other cereals, vegetable and fruit, kg), *Rat_straw_* refers to ratio of straw to grain (%)，*Pcon_straw_* refers to P contents in straw(%). *Rat_straw-field_* refers to ratio of straw returning to field, $\text{NU}\text{ani}$ refers to animal numbers of different kinds of animal (pig, dairy cattle, beef cattle, meat poultry, sheep and goat, livestock unit (LU)

), *Pex_ani_* is P excretion per animal per year of different animals (kg/unit).

| $\text{PO}\text{le}\text{=(}\text{PI}\text{fer}\text{-PO}\text{main-crop}\text{-PO}\text{by-crop}\text{-PO}\text{rf}\text{)}\text{×}\text{EF}\text{leaching}$ | $\text{（}\text{8}\text{）}$ |
| --- | --- |
| $\text{PO}\text{main-crop}$*=Prod_crop_*$\text{×}$*Pcon_crop_* | $\text{（}\text{9}\text{）}$ |
| $\text{PO}\text{by-crop}$*=Prod_crop_*$\text{×}$*Rat_straw_*$\text{×}$*Pcon_straw_* | $\text{（}\text{10}\text{）}$ |

Where, $\text{PO}\text{main-crop}$ refers to output of P via main crop products from crop production, *Pcon_crop_* is P contents in crop products (%), $\text{PO}\text{by-crop}$ refers to output of P via by-products from crop production. $\text{EF}\text{leaching}$ is emission factor of P from crop production by leaching.

| $\text{PO}\text{manure-le}\text{=}\text{NU}\text{ani}\text{×Pex}\text{ani}\text{×EF}\text{am-leaching}$ | $\text{（}\text{11}\text{）}$ |
| --- | --- |

$\text{NU}\text{ani}$ refers to animal numbers of different kinds of animal (pig, dairy cattle, beef cattle, meat poultry, sheep and goat, livestock unit),$\text{EF}\text{am-leaching}$ is emission factor of P from animal production by animal manure leaching during storage (%).

| $\text{ }\text{PO}\text{soil}\text{=}\text{(}\text{PI}\text{fe}\text{+PI}\text{st}\text{+PI}\text{am}\text{-}\text{PO}\text{main-crop}\text{-PO}\text{by-crop}\text{-}\text{PO}\text{rf}\text{)}\text{×}\text{EF}\text{accumulate}\text{ }$ | $\text{（}\text{12}\text{）}$ |
| --- | --- |

$\text{EF}\text{accumulate}$ refers to emission factor of P from crop production by soil accumulation (%)。

Table S1 sources of the data for model inputs. The abbreviations are explained in the main text “Method”

| model inputs | Units | Source | Scale | Value |
| --- | --- | --- | --- | --- |
| $\text{PI}\text{fer}$ | kg | Yunnan statistical yearbook | County | Table.S2 and S3 |
| $\text{PI}\text{if}$ | kg | Yunnan statistical yearbook | County | Table.S3 |
| *Prod_crop_* | kg | Yunnan statistical yearbook | County | Table.S2 and S3 |
| $\text{NU}\text{ani}$ | LU | Yunnan statistical yearbook | County | Table.S4and S5 |
| *Pcon_crop_* | g/kg | Ma et al (2010),Zhao et al(2017), Wang et al(2018) | Province. County | Table.S6 |
| *Pcon_straw_* | g/kg | Ma et al (2010) | Province | Table.S6 |
| *Rat_straw_* | 0-1,fraction | Ma et al (2010) | Province | 0.08 |
| *Rat_straw-field_* | 0-1,fraction | field surveys and direct interactions with local experts | Village | 0.3 |
| *Pex_ani_* | kg/LU | Ma et al (2010),Zhao et al(2017), Wang et al(2018) | Province. County | Table.S7 |
| *EF_runoff and erossion_* | 0-1,fraction | Ma et al (2010),Zhao et al(2017), Wang et al(2018) | County | Table.S8 and S9 |
| $\text{EF}\text{leaching}$ | 0-1,fraction | Li et al (2018) | County | Table.S8 and S9 |
| $\text{EF}\text{am-leaching}$ | 0-1,fraction | Ma et al (2010),Zhao et al(2017), Wang et al(2018) | County | Table.S10 |
| $\text{EF}\text{accumulate}\text{ }$ | 0-1,fraction | Ma et al (2010),Zhao et al(2017), Wang et al(2018) | County | Table.S8 and S9 |

* Livetock Unit (LU)= a cattle (600kg) (mean values with their ranges in parentheses)

Table S2 The application of synthetic fertilizer, cereals production(yield), vegetables production(yield), fruit production(yield) and feed import in Yunnan province from 1995 to 2014.

| Model input | 1995 | 1996 | 1997 | 1998 | 1999 | 2000 | 2001 | 2002 | 2003 | 2004 | 2005 | 2006 | 2007 | 2008 | 2009 | 2010 | 2011 | 2012 | 2013 | 2014 |
| --- | --- | --- | --- | --- | --- | --- | --- | --- | --- | --- | --- | --- | --- | --- | --- | --- | --- | --- | --- | --- |
| Synthetic fertilizer(10^8^kg) | 1.58 | 1.69 | 1.75 | 1.76 | 1.8 | 1.83 | 1.99 | 2.17 | 2.15 | 2.27 | 2.27 | 2.38 | 2.48 | 2.6 | 2.54 | 2.72 | 2.96 | 3.06 | 3.21 | 3.34 |
| Cereals production(10^10^kg) | 1.19 | 1.25 | 1.27 | 1.32 | 1.4 | 1.47 | 1.49 | 1.42 | 1.47 | 1.51 | 1.51 | 1.46 | 1.46 | 1.52 | 1.58 | 1.53 | 1.67 | 1.75 | 1.82 | 1.86 |
| Vegetables production(10^10^kg) | 0.41 | 0.43 | 0.5 | 0.51 | 0.53 | 0.59 | 0.65 | 0.75 | 0.84 | 0.89 | 0.97 | 1.03 | 1.11 | 1.17 | 1.24 | 1.26 | 1.3 | 1.47 | 1.63 | 1.74 |
| Fruit production(10^9^kg) | 0.56 | 0.59 | 0.66 | 0.68 | 0.74 | 0.77 | 0.98 | 0.86 | 0.97 | 1.16 | 1.37 | 1.63 | 2.02 | 2.66 | 3.04 | 3.41 | 4.05 | 5.11 | 5.11 | 5.11 |
| Cereals yield(10^5^kg/km^2^) | 3.26 | 3.37 | 3.42 | 3.40 | 3.46 | 3.46 | 3.43 | 3.42 | 3.62 | 3.63 | 3.56 | 3.62 | 3.66 | 3.71 | 3.75 | 3.58 | 3.87 | 3.98 | 4.05 | 4.13 |
| Vegetables yield(10^6^kg/km^2^) | 1.93 | 1.96 | 2.21 | 2.08 | 1.81 | 1.67 | 1.67 | 1.86 | 1.92 | 1.92 | 1.97 | 1.98 | 1.98 | 2.00 | 1.99 | 1.87 | 1.82 | 1.83 | 1.80 | 1.83 |
| Fruit yield(10^5^kg/km^2^) | 3.87 | 3.19 | 3.63 | 3.49 | 3.70 | 3.36 | 4.48 | 4.04 | 4.43 | 5.12 | 6.08 | 6.72 | 7.63 | 9.21 | 9.82 | 10.84 | 11.63 | 13.01 | 12.56 | 12.56 |
| Feed import(10^5^kg) | 1.59 | 1.55 | 1.58 | 1.60 | 1.63 | 1.70 | 1.58 | 1.52 | 1.54 | 1.58 | 1.59 | 1.42 | 1.44 | 1.44 | 1.49 | 1.51 | 1.46 | 1.44 | 1.37 | 1.40 |

Table S3 The application of synthetic fertilizer, cereals production(yield), vegetables production(yield), fruit production(yield) and feed import in 16 municipalities of Yunnan province in 2014.

| Model input | Kunming | Qujing | Yuxi | Baoshan | Zhaotong | Lijiang | Pu’er | Lincang | Chuxiong | Honghe | Wenshan | Xishuangbanna | Dali | Dehong | Nujiang | Diqing |
| --- | --- | --- | --- | --- | --- | --- | --- | --- | --- | --- | --- | --- | --- | --- | --- | --- |
| Synthetic fertilizer(10^7^kg) | 3.72 | 7.86 | 1.16 | 1.58 | 2.04 | 2.17 | 0.75 | 0.74 | 2.84 | 3.82 | 2.42 | 0.57 | 2.62 | 0.76 | 0.08 | 0.29 |
| Cereals production(10^10^kg) | 1.19 | 1.25 | 1.27 | 1.32 | 1.4 | 1.47 | 1.49 | 1.42 | 1.47 | 1.51 | 1.51 | 1.46 | 1.46 | 1.52 | 1.58 | 1.53 |
| Vegetables production(10^10^kg) | 0.41 | 0.43 | 0.5 | 0.51 | 0.53 | 0.59 | 0.65 | 0.75 | 0.84 | 0.89 | 0.97 | 1.03 | 1.11 | 1.17 | 1.24 | 1.26 |
| Fruit production(10^9^kg) | 0.56 | 0.59 | 0.66 | 0.68 | 0.74 | 0.77 | 0.98 | 0.86 | 0.97 | 1.16 | 1.37 | 1.63 | 2.02 | 2.66 | 3.04 | 3.41 |
| Cereals yield(10^3^kg/km^2^) | 4.55 | 4.86 | 5.54 | 0.54 | 4.03 | 3.84 | 3.33 | 3.45 | 4.85 | 4.54 | 3.41 | 5.41 | 5.55 | 4.92 | 2.51 | 3.67 |
| Vegetables yield(10^4^kg/km^2^) | 2.84 | 1.63 | 2.60 | 1.84 | 1.51 | 1.98 | 0.82 | 2.18 | 2.35 | 2.27 | 0.70 | 1.11 | 2.68 | 1.22 | 0.83 | 1.82 |
| Fruit yield(10^4^kg/km^2^) | 1.12 | 0.37 | 2.30 | 0.27 | 1.56 | 1.30 | 0.89 | 0.49 | 0.98 | 6.39 | 0.65 | 14.59 | 3.22 | 0.29 | 0.18 | 0.48 |
| Feed import(10^3^kg) | 26.1 | 35.8 | 7.0 | 13.0 | 7.0 | 8.3 | 8.9 | 7.8 | 15.6 | 26.0 | 15.3 | 0.6 | 20.6 | 1.3 | 2.6 | 1.2 |

Table S4 Animal number of pig, meat cow, cattle, poultry and sheep in Yunnan province from 1995 to 2014.

| Model input | 1995 | 1996 | 1997 | 1998 | 1999 | 2000 | 2001 | 2002 | 2003 | 2004 | 2005 | 2006 | 2007 | 2008 | 2009 | 2010 | 2011 | 2012 | 2013 | 2014 |
| --- | --- | --- | --- | --- | --- | --- | --- | --- | --- | --- | --- | --- | --- | --- | --- | --- | --- | --- | --- | --- |
| Pig (×10^6^LU) | 6.89 | 7.30 | 7.38 | 7.52 | 7.38 | 7.76 | 7.56 | 7.46 | 7.66 | 7.82 | 7.81 | 7.10 | 7.37 | 8.01 | 8.21 | 8.30 | 8.07 | 8.13 | 8.13 | 8.04 |
| Beef cattle (×10^6^LU) | 6.29 | 6.02 | 6.12 | 6.24 | 6.55 | 6.84 | 6.35 | 6.00 | 6.10 | 6.31 | 6.42 | 5.70 | 5.81 | 5.65 | 5.94 | 5.97 | 5.97 | 5.98 | 5.84 | 6.01 |
| Cattle (×10^6^LU) | 7.73 | 7.43 | 7.58 | 7.66 | 8.00 | 8.29 | 7.79 | 7.44 | 7.53 | 7.73 | 7.50 | 7.14 | 7.22 | 7.07 | 7.36 | 7.38 | 7.42 | 7.41 | 7.05 | 7.38 |
| Poultry(×10^6^LU) | 0.65 | 0.71 | 0.79 | 0.93 | 1.03 | 1.16 | 1.25 | 1.35 | 1.50 | 1.60 | 1.70 | 1.94 | 2.09 | 2.32 | 2.51 | 2.70 | 2.73 | 2.86 | 2.80 | 2.76 |
| Sheep(×10^5^LU) | 7.19 | 7.34 | 7.68 | 8.01 | 8.36 | 8.93 | 8.38 | 7.84 | 8.10 | 8.53 | 9.77 | 7.93 | 8.26 | 8.43 | 8.78 | 8.78 | 9.01 | 9.14 | 9.29 | 10.08 |

Table S5 Animal number of pig, beef cattle, other cattle, poultry and sheep in 16 municipalities of Yunnan province in 2014.

| Model input | Kunming | Qujing | Yuxi | Baoshan | Zhaotong | Lijiang | Pu’er | Lincang | Chuxiong | Honghe | Wenshan | Xishuangbanna | Dali | Dehong | Nujiang | Diqing |
| --- | --- | --- | --- | --- | --- | --- | --- | --- | --- | --- | --- | --- | --- | --- | --- | --- |
| Pig (×10^6^LU) | 1.31 | 4.80 | 0.69 | 1.56 | 1.35 | 0.50 | 0.67 | 1.04 | 1.09 | 2.52 | 1.82 | 0.13 | 1.37 | 0.27 | 0.13 | 0.10 |
| Beef cattle (×10^5^LU) | 2.95 | 7.58 | 1.93 | 1.88 | 2.22 | 0.94 | 5.48 | 1.80 | 2.91 | 3.68 | 5.38 | 0.32 | 4.80 | 0.84 | 0.28 | 0.34 |
| Other cattle (×10^5^LU) | 6.90 | \ | \ | \ | \ | 4.88 | \ | \ | 8.19 | 10.79 | 0.29 | 0.54 | 10.86 | \ | 1.66 | 0.32 |
| Poultry(×10^6^LU) | 4.63 | 0.51 | 0.54 | 0.16 | 0.17 | 0.05 | 0.19 | 0.21 | 0.30 | 0.73 | 0.26 | 0.06 | 0.31 | 0.11 | 0.03 | 0.01 |
| Sheep(×10^5^LU) | 1.45 | 3.29 | 0.45 | 0.76 | 0.48 | 1.29 | 0.44 | 0.75 | 1.57 | 1.00 | 0.45 | 0.02 | 1.63 | 0.09 | 0.49 | 0.22 |

Note:\ refers to no data.

Table S6 Ratio of straw to grain and P contents in crop products and straw

| Crop | *Pcon_straw_* (g/kg) | *Pcon_crop_* (g/kg) | |
| --- | --- | --- | --- |
|  |  | Grain | Straw |
| Maize | 1.2 | 4.3 | 1.3 |
| Wheat | 1.1 | 3.7 | 0.7 |
| Milet | 1.6 | 4.7 | 0.9 |
| Other cereals | 1.6 | 5.1 | 1.3 |
| Vegetable | 0 | 0.6 | — |
| Fruit | 0 | 1 | — |

Table S7 P excretion per animal per year of different animals

| Animal category | *Pex_ani_*（kg/LU） |
| --- | --- |
| Pig | 1.69 |
| Dairy cattle | 12.92 |
| Beef cattle | 4.84 |
| Draught cattle | 5.13 |
| Sheep and goats | 1.03 |
| Meat poultry | 0.02 |

**Table S8 P content of production in animal body**

| Animal | Fresh weight(kg) | Partition of animal live weight (%) | | | P content (g/kg) | | |
| --- | --- | --- | --- | --- | --- | --- | --- |
|  |  | Edible part | Bone | Other parts | Edible part | Bone | Other parts |
| Pig | 90 | 50 | 13 | 37 | 1.8 | 33 | 0.7 |
| Dairy cattle | — | 100 | — | — | 0.9 | — | — |
| Beef cattle | 477.3 | 45 | 20 | 35 | 1.7 | 42 | 0.1 |
| Sheep and goats | 35 | 20 | 24 | 21 | 1.7 | 56 | 1.5 |
| Poultry | 2.04 | 65 | 20 | 15 | 1.6 | 20 | 0.1 |
| Eggs | — | 100 | — | — | 2.1 | — | — |

—: no data

Table S9 Emission factors of runoff and erosion, leaching and soil accumulation in Yunnan province from 1995 to 2014

| Parameters | 1995 | 1996 | 1997 | 1998 | 1999 | 2000 | 2001 | 2002 | 2003 | 2004 | 2005 | 2006 | 2007 | 2008 | 2009 | 2010 | 2011 | 2012 | 2013 | 2014 |
| --- | --- | --- | --- | --- | --- | --- | --- | --- | --- | --- | --- | --- | --- | --- | --- | --- | --- | --- | --- | --- |
| *EF_runoff and erossion_* (%) | 16.2 | 16.2 | 16.2 | 16.2 | 16.2 | 16.2 | 16.2 | 16.2 | 16.2 | 16.2 | 16.2 | 16.2 | 16.2 | 16.2 | 16.2 | 16.2 | 16.2 | 16.2 | 16.2 | 16.2 |
| $\text{EF}\text{leaching}$ (%) | 6.29 | 6.02 | 6.12 | 6.24 | 6.55 | 6.84 | 6.35 | 6.00 | 6.10 | 6.31 | 6.42 | 5.70 | 5.81 | 5.65 | 5.94 | 5.97 | 5.97 | 5.98 | 5.84 | 6.01 |
| $\text{EF}\text{accumulate}\text{ }$(%) | 7.73 | 7.43 | 7.58 | 7.66 | 8.00 | 8.29 | 7.79 | 7.44 | 7.53 | 7.73 | 7.50 | 7.14 | 7.22 | 7.07 | 7.36 | 7.38 | 7.42 | 7.41 | 7.05 | 7.38 |

Table S10 Emission factors of runoff and erosion, leaching and soil accumulation in 16 municipalities of Yunnan province in 2014

| Parameters | Kunming | Qujing | Yuxi | Baoshan | Zhaotong | Lijiang | Pu’er | Lincang | Chuxiong | Honghe | Wenshan | Xishuangbanna | Dali | Dehong | Nujiang | Diqing |
| --- | --- | --- | --- | --- | --- | --- | --- | --- | --- | --- | --- | --- | --- | --- | --- | --- |
| *EF_runoff and erossion_* (%) | 11.80 | 11.80 | 13.15 | 13.15 | 13.15 | 13.15 | 13.15 | 13.15 | 13.15 | 13.15 | 13.15 | 11.80 | 13.15 | 11.80 | 14.50 | 14.50 |
| $\text{EF}\text{leaching}$ (%) | 0.80 | 1.20 | 0.80 | 0.80 | 0.80 | 0.80 | 0.80 | 0.80 | 0.80 | 0.80 | 0.80 | 0.80 | 0.80 | 0.80 | 0.80 | 1.20 |
| $\text{EF}\text{accumulate}\text{ }$(%) | 99.20 | 98.80 | 99.20 | 99.20 | 99.20 | 99.20 | 99.20 | 99.20 | 99.20 | 99.20 | 99.20 | 99.20 | 99.20 | 99.20 | 98.80 | 99.89 |

Table S11 Emission factors of manure leaching during storage in Yunnan province from 1995 to 2014 ($\text{EF}\text{am-leaching}$(%))

| Parameters | 1995 | 1996 | 1997 | 1998 | 1999 | 2000 | 2001 | 2002 | 2003 | 2004 | 2005 | 2006 | 2007 | 2008 | 2009 | 2010 | 2011 | 2012 | 2013 | 2014 |
| --- | --- | --- | --- | --- | --- | --- | --- | --- | --- | --- | --- | --- | --- | --- | --- | --- | --- | --- | --- | --- |
| Pig | 9 | 11 | 13 | 15 | 16 | 17 | 19 | 23 | 25 | 26 | 27 | 28 | 29 | 30 | 31 | 33 | 35 | 36 | 37 | 39 |
| Beef cattle | 2.7 | 3 | 4 | 5 | 6 | 7 | 8 | 9 | 11 | 13 | 15 | 15 | 16 | 15 | 17 | 18 | 19 | 21 | 22 | 23 |
| Poultry | 10 | 12 | 14 | 17 | 20 | 21 | 24 | 26 | 27 | 28 | 29 | 30 | 31 | 32 | 34 | 36 | 38 | 40 | 42 | 44 |
| Other cattle | 2.7 | 3 | 4 | 5 | 6 | 7 | 8 | 9 | 11 | 13 | 15 | 15 | 16 | 15 | 17 | 18 | 19 | 21 | 22 | 23 |
| Sheep | 12 | 14 | 16 | 19 | 21 | 24 | 25 | 26 | 27 | 28 | 29 | 30 | 31 | 32 | 34 | 36 | 38 | 40 | 42 | 44 |

A.2 Data sources

Three main data sources were used for deriving input data of SFA model.

1.Yunnan Statistical Yearbook: An authoritative statistical source documenting fertilizer use, crop production, crop yield, imported feed, and livestock numbers at the county level. Fertilizer application was determined based on inputs from both single and compound fertilizers. Crop production and yield data covered three major crop categories—cereals, vegetables, and fruits—at the county level. The sown areas of these three crops accounted for more than 80% of the total cultivated land. Livestock data included five categories: pigs, beef cattle, other cattle, poultry, and sheep.

2.Literature Data: Information on phosphorus (P) concentration in harvested crops and animal products, P excretion values per livestock category, and the partitioning of animal products into edible and non-edible parts. Most of these values were obtained from measurements and were not regionally differentiated. Additionally, phosphorus leaching, runoff, and erosion into surface water can occur due to uncovered and unsealed manure storage and processing systems, direct discharge of animal manure, and agricultural land runoff. Transfer coefficients and emission factors were estimated based on factors such as temperature, slope, soil texture, land use, and rainfall (Ma et al., 2010). Consequently, phosphorus loss pathways varied significantly across regions due to differences in climate, soil conditions, nutrient application, land use, and crop management practices.

3.Farm Surveys and Local Experts: The farm survey was conducted using questionnaires and provided insights into farm structure and management practices. A stratified random sampling approach was used to select survey areas and households. In total, 1,205 farmers across 12 counties in 16 municipalities were interviewed regarding farm structure, synthetic fertilizer use, livestock farming, and manure management. The collected questionnaire data were processed and statistically analyzed, with approximately 10% of responses discarded due to inconsistencies or illogical data. Additionally, certain model parameters were derived from consultations with local experts in China, including those from Southwest Forestry University and China Agricultural University.

A.3 Spatial changes of crop production and animal production

Fig.S1a illustrates the crop sown area and the proportion of different kinds of crops across 16 municipalities in Yunnan Province in 1995. Based on the total sown ares, Yunnan Province can be divided into three regions: the first region (<30×10^4^ha) includes Dehong, Nujiang, Diqing, Lijiang, Yuxi, and Xishuangbanna; the second region (30-60×10^4^ha) includes Pu'er, Lincang, Baoshan, Dali, Chuxiong, and Kunming; and the third region (>60×10^4^ha) includes Honghe, Wenshan, Qujing, and Zhaotong. In terms of crop types, all 16municipalities in Yunnan Province primarily focused on cereals crop cultivation, accounting for more than 50% of the total sown area. Among them, Nujiang, Diqing, and Lijiang were the main cereals crop-producing regions, accounting for 89%, 87%, and 86% of the total sown area, respectively. Figure.1Sb depicts the crop sown area and the proportion of various major crops across 16 municipalities in Yunnan Province in 2014. Compared to 1995, the sown area of crops in Wenshan and Honghe increased significantly. In terms of crop types, although most municipalities remained focused on cereal crop cultivation, the proportion of vegetable cultivation in Yuxi, Kunming, and Chuxiong increased to 18%, 20%, and 28% of the total sown area, respectively. Additionally, Dehong and Lincang emerged as major fruit-producing regions, with fruit cultivation accounting for 37% and 33% of the total sown area, respectively.

**
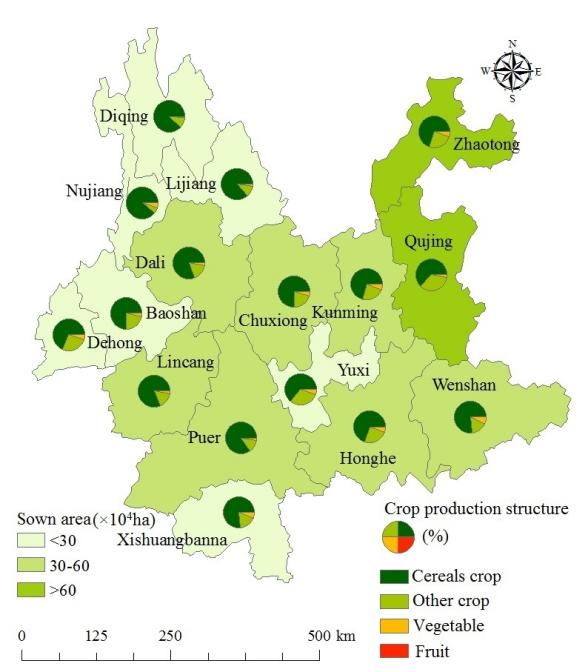

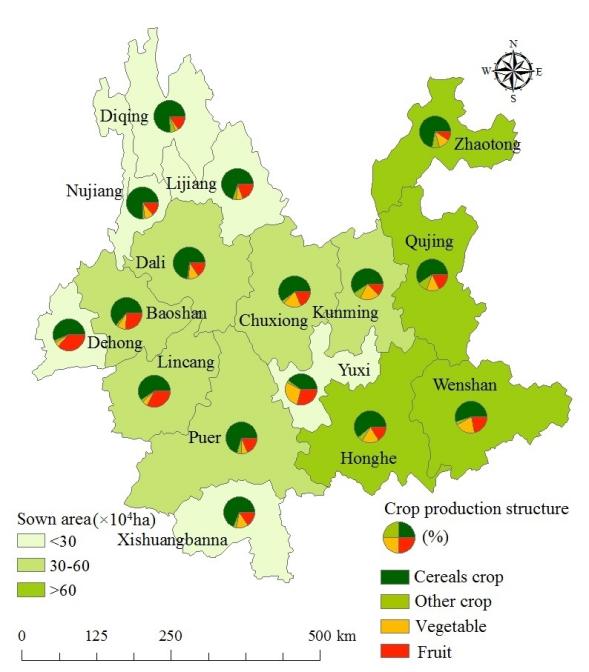
**

b

a

**Fig.S1** **Spatial changes of crop production structures of Yunnan in 1995(a) and 2014(b)**

To facilitate comparison of the numbers of different animal, we used Livestock Unit (LU) to quantify the number of animals. Fig.S2a illustrates the changes of number of animals across the 16 municipalities of Yunnan Province in 1995, along with the proportion of major livestock types. Based on the number of animals, Yunnan Province can be divided into three regions: the first region (<100×10⁴ LU) includes Diqing, Nujiang, Dehong, and Xishuangbanna; the second region (100-200×10⁴ LU) includes Lijiang, Baoshan, Lincang, Pu'er, Yuxi, and Zhaotong; and the third region (>200×10⁴ LU) includes Dali, Chuxiong, Kunming, Qujing, Wenshan, and Dehong. Among these, Kunming primarily focuses on poultry production, accounting for 44%. Fig.S2b shows the changes of number of animals across the 16 municipalities of Yunnan Province in 2014, along with the proportion of major livestock types. Compared to 1995, there was no significant change in the total number of animals across the municipalities in 2014. In terms of livestock types, Kunming continued to focus on poultry production. Additionally, the proportion of pigs raised in Baoshan, Qujing, Lincang, and Wenshan increased, reaching by 78%, 75%, 69%, and 67%, respectively.


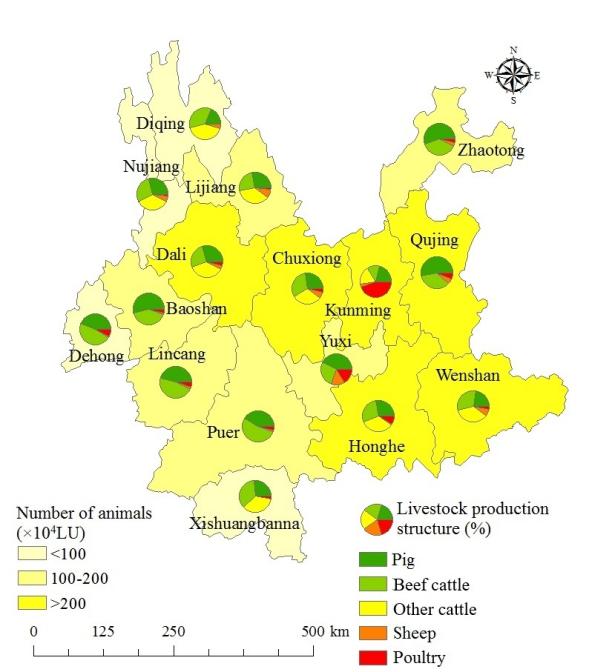
**
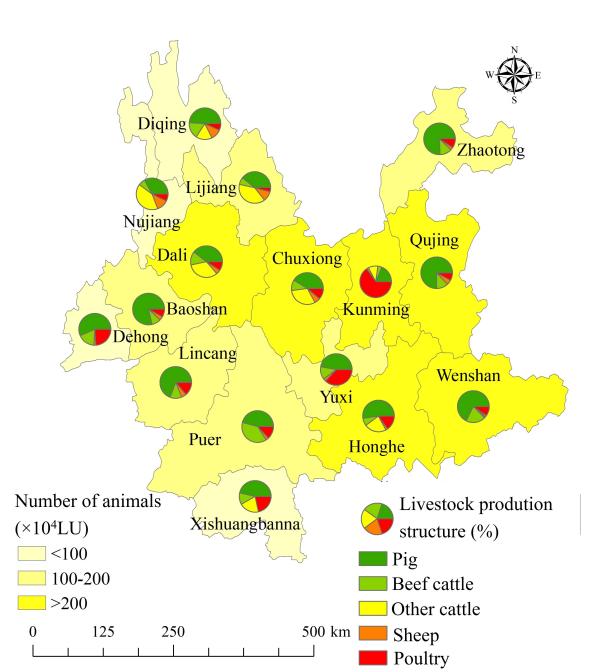
**

b

a

**Fig.S2 Spatial changes of animal production structures of Yunnan in 1995 (a) and 2014(b)**

Table.S12 Summary of municipality-level PUE value and risk categories in Yunnan province

| code | Municipality | PUE | Risk categories |
| --- | --- | --- | --- |
| 1 | Kunming | 15.7 | RegionⅠ |
| 2 | Qujing | 18.9 | RegionⅠ |
| 3 | Yuxi | 28.7 | RegionⅡ |
| 4 | Baoshan | 5.0 | RegionⅠ |
| 5 | Zhaotong | 47.5 | RegionⅢ |
| 6 | Lijiang | 10.9 | RegionⅠ |
| 7 | Pu’er | 50.7 | RegionⅢ |
| 8 | Lincang | 47.0 | RegionⅢ |
| 9 | Chuxiong | 19.7 | RegionⅠ |
| 10 | Honghe | 21.1 | RegionⅠ |
| 11 | Wenshan | 27.3 | RegionⅡ |
| 12 | Xishuangbanna | 43.8 | RegionⅢ |
| 13 | Dali | 25.4 | RegionⅡ |
| 14 | Dehong | 46.8 | RegionⅢ |
| 15 | Nujiang | 44.3 | RegionⅢ |
| 16 | Diqing | 26.6 | RegionⅡ |
